# Amnestic MCI Patients’ Perspectives toward Disclosure of Amyloid PET Results in a Research Context

**DOI:** 10.1007/s12152-017-9313-z

**Published:** 2017-03-21

**Authors:** Gwendolien Vanderschaeghe, Jolien Schaeverbeke, Rik Vandenberghe, Kris Dierickx

**Affiliations:** 1 Centre for Biomedical Ethics and Law, Department of Public Health and Primary Care, KU Leuven, Kapucijnenvoer 35 Blok D Box 7001, 3000 Leuven, Belgium; 2 Laboratory for Cognitive Neurology, Department of Neurosciences, KU Leuven, 0&N II, Herestraat 49 box 1027, 3000 Leuven, Belgium; 3 Alzheimer Research Centre KU Leuven, Leuven research Institute for Neurodegenerative Disorders, Leuven, Belgium; 4 Memory Clinic / Neurology, University Hospitals Leuven (UZ Leuven, Campus Gasthuisberg), Leuven, Belgium

**Keywords:** Ethics, Amnestic MCI, Clinical trial, Qualitative research, Disclosure of return of results, Individual research results, Biomarker, Amyloid PET, Belgium

## Abstract

**Background:**

Researchers currently are not obligated to share individual research results (IRR) with participants. This non-disclosure policy has been challenged on the basis of participants’ rights to be aware and in control of their personal medical information. Here, we determined how patients view disclosure of research PET results of brain amyloid and why they believe it is advantageous or disadvantageous to disclose.

**Method:**

As a part of a larger diagnostic trial, we conducted semi-structured interviews with patients with amnestic Mild Cognitive Impairment (aMCI). Participants had the option to receive their brain amyloid PET scan result (i.e., their IRR). Interviews were conducted before they received their IRR.

**Results:**

A total of 38 aMCI patients (100% of study participants) wanted to know their IRR. The two most frequently mentioned reasons for choosing IRR disclosure were to better understand their brain health status and to be better able to make informed decisions about future personal arrangements (e.g., inheritance tax, moving into a smaller house, end-of-life decisions, etc.). Emotional risk was mentioned as the primary disadvantage of knowing one’s IRR. On the other hand, non-disclosure was considered to be emotionally difficult also, as patients would be uncertain about their future health condition.

**Conclusions:**

Many patients diagnosed clinically with aMCI want to know their brain amyloid test results, even though this knowledge may be disadvantageous to them. Knowing what is going on with their health and the ability to make informed decisions about their future were the two principal advantages mentioned for obtaining their amyloid PET results. Because of the overwhelming consensus of aMCI patients was to disclose their brain amyloid PET scan results, researchers should strongly consider releasing this information to research subjects.

## Background

Biomarkers are biological indicators that can gauge the presence of a disease and can be used to support clinical diagnosis [1]. One of the biomarkers used to support a diagnosis of Alzheimer’s Disease (AD) is based on brain amyloid, which can be visualized in situ by positron emission tomography (PET) and radiotracers that have a high affinity for binding to beta-amyloid plaques in vivo. PET imaging of beta-amyloid plaques in the brain has been approved for diagnostic purposes in cognitively impaired patients who are being evaluated for AD [2, 3]. Besides being useful for clinical situations, PET imaging of beta-amyloid plaques is also being used in brain research on AD.

Little is known about patients’ motivations, opinions, and experiences in relation to their clinical trial participation and their IRR. In a research context, the degree to which individual research results (IRR) are shared with study participants varies, as the researcher is not obligated to do so [3, 4]. Reasons to support the non-disclosure policy mostly relate to differences between the research and clinical context, the limited efficacy of current treatments, the experimental nature of certain types of data, and the limitations of lay participants to objectively and reasonably interpret the results [5, 6]. On the other hand, some reasons to support IRR disclosure are the right of participants to know and be in control of information about their medical condition, it affords participants the opportunity to make informed lifestyle changes and practical personal and family arrangements, and it opens up the possibility for participants to enroll in (early) medical interventions [5, 7, 8]. The previously mentioned reasons are based on the available theoretical literature and are mostly used by researchers to support or withhold IRR disclosure. Yet, what are the concrete reasons and motivations of research participants? Thus, the following questions arise. Why would participants opt for the possibility to be informed about their IRR? What do participants perceive as possible advantages and disadvantages of knowing their IRR? Clear answers to these questions will help guide researchers and experimental subjects in deciding which option—IRR disclosure or non-disclosure—to choose.

In this study, we focused on the reasons why patients with amnestic mild cognitive impairment (aMCI) were either in favor of or against the disclosure of their brain amyloid PET results. The experiences and opinions of these aMCI patients can differ from those of healthy individuals being screened for AD [9]. We also focused on the disclosure of brain amyloid PET scan results, because these biomarker findings have been approved by the FDA (Food and Drug Administration) and EMA (European Medicines Agency) for clinical use in patients with a cognitive deficit [10–12]. Our findings are based on interviews with aMCI patients before they received their IRR.

## Methods

### Recruitment

Recruitment took place between June 2015 and June 2016 after approval of the study by the Ethics Committee, University Hospitals Leuven. All participants provided written informed consent in accordance with the Declaration of Helsinki.

The study cohort consisted of a consecutive series of aMCI patients [13] recruited via the memory clinic of the University Hospitals Leuven. The interview was part of a sub-study of the BioAdaptAD study, an investigator-driven longitudinal study of aMCI. The primary objective of the BioAdaptAD study (EUDRACT no. 2013–004671-12) was to evaluate the predictive value of baseline amyloid biomarker measurements for tracking longitudinal cognitive change over a two-year period. When candidate subjects met the inclusion criteria (see Appendix 1) of the BioAdaptAD study, they were given the option to participate in a sub-study investigating the opportunities to receive their IRR and the ethical challenges associated with it. More specifically, this sub-study provided the participants with the option of receiving their amyloid PET scan (binary visual read or positive/negative for brain amyloid) results that were obtained within the context of the BioAdaptAD study. We conducted semi-structured interviews with participants who agreed to take part in the sub-study in order to better understand their motivations, opinions, and experiences about the disclosure of their IRR.

Before the start of the sub-study, an informed consent brochure was given to candidate subjects. The content of this brochure was based on the E6 Guideline of Good Clinical Practice and contained background information about the study, study objectives, the interview process, and research subjects’ rights. Before the scheduled interview, the interviewer orally repeated the content of the informed consent brochure and asked the candidate whether they had any further questions. If they hesitated or had doubts about participating, the interview was re-scheduled to a later time to give the candidate sufficient time to decide.

### Data Collection and Analysis

The interview guide was developed by GV, KD, and RV, and its content was based on findings in the literature on the topic of IRR. The first two interviews constituted a pilot study, which was used to evaluate the interview guide and make needed adjustments. The interview questions covered three content areas. The first part of the interview consisted of questions intended to help us better understand how patients describe and experience their current memory problems. Although patients’ description of their memory complaints were not intended to be part of the result section of this manuscript, these exploratory questions were used as opening questions for the interview and to provide the interviewer with some concrete patients’ experiences regarding their memory complaints. The second part consisted of open-ended questions about why they want to know their IRR result and what they perceive as possible (dis)advantages of their IRR disclosure. The third part consisted of hypothetical questions about how the patient thinks he would respond to a set of possible situations, such as: ‘What if the researcher informs you that you have a positive amyloid PET scan result? How do you think you will respond to this news? Several interview techniques were used, such as rephrasing part of the participant’s answer, asking yes or no questions, to briefly check whether the interviewer understood the participant’s answer correctly. Supplementary questions were also asked to get more in-depth information from the participant.

After a short introduction of what the research was all about and what to expect in the interviews, patients were invited to sign the informed consent form (IC) and then to complete the sociodemographic information form. Completion of the IC form indicated that the patient understood that his/her results and interview records would remain confidential, that participation in the interview was voluntary, and that it would have no impact on his/her participation in the general study or on any other medical intervention he/she might undergo at the hospital. Participants were informed that results of the study would be published in a scientific journal and that a lay description of results from the interviews would be provided to them after completion of the study.

The interviews were recorded on tape with the consent of the interviewee. A mixed-method approach was used to analyze the interviews. (1) Transcripts were analyzed using QSR International’s Nvivo 10 software and was performed according to qualitative conventional content analysis methodology [14, 15]. In the first phase, the interviewer (GV) coded the interviews three separate times, with an interval of a few weeks between each coding session. Letting time pass between each session and re-coding allows one to check whether new interpretations might be attributed to the content that could have been missed in just one coding session. To protect against bias, in the second analysis phase, we had five interviews independently coded by a second researcher. In the final phase, we compared the codes assigned by the interviewer and those done by the independent researcher (KD), working to reach a consensus on the final codes to be used. (2) In addition, we used a quantitative approach to analyze the reasons, benefits, and disadvantages of receiving an IRR, as provided by the participants.

Interviews were conducted in Dutch, with the exception of one, which was conducted in English. For this interview, the patient preferred to speak in his native language, English. Quotations of patients presented here have been translated into English.

## Results

### Study Population

The study population consisted of 38 aMCI patients who met the inclusion criteria of the study. Table 1 summarizes the demographic information and scores of the neuropsychological evaluation of the study population.

**Table 1:** Demographic Characteristics and neuropsychological evaluation scores of the study population of aMCI patients

Characteristics	*n =* number
Mean age:	
71 yr. ± 6.5 (range 55–83 yr)	-
Gender:	
Male	22
Female	16
Highest educational level attained:	
Primary school	7
Secondary school	17
Professional bachelor	6
Academic master	8
Marital status:	
Married	30
Widow/widower	4
Divorced	4
≥1 Child(ren)	36
Neuropsychological evaluation	Mean score
Global Clinical Dementia Rating (CDR)	0.5
Mini Mental State Examination (MMSE)	27.8 (range: 25–30)
Auditory Verbal Learning Test (AVLT) Total Learning (/75)	36.2 + − 10.2
AVLT long term % recall	61.7% + − 29.6%

### Reasons for Wanting to Know Brain Amyloid PET Scan Result (IRR)

**Fig. 1:**
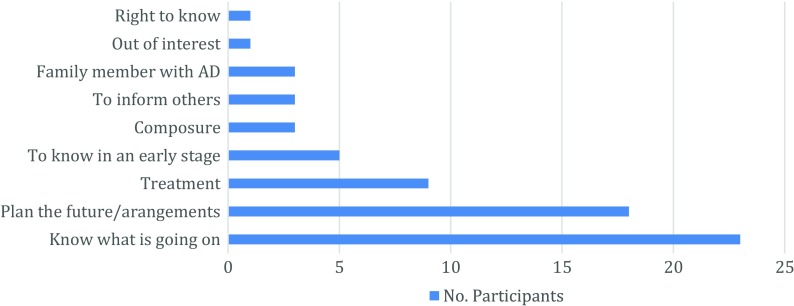
Frequency of reasons participants mentioned for wanting to know their brain amyloid PET scan result (i.e., IRR).*

The results presented here are based on participants’ answers in the interview *before* receiving their IRR. We decided to conduct the interviews in this order so that we could get unbiased answers as to why patients might want to know their IRR. All participants expressed the desire to know their individual brain amyloid PET scan result (i.e., IRR). Although one participant initially did not want to know his IRR, after thinking more about it, he changed his mind. Overall, nine reasons were mentioned by the participants for wanting to know their IRR. Some participants mentioned two or three reasons (Fig. 1). These reasons will be considered in turn and elaborations will be provided in participants’ quotes.

**Fig. 2:**
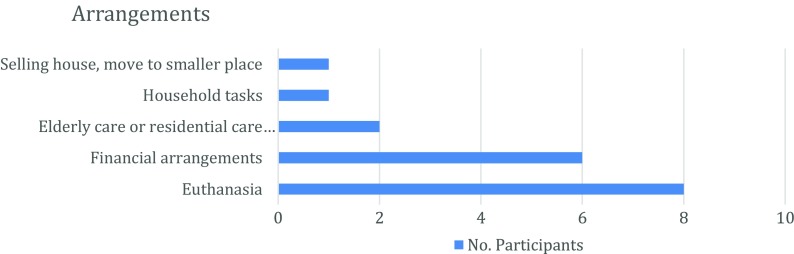
Frequencies of topical references made in relation to IRR affording the opportunity for making arrangements or planning for the future

* Some participants mentioned more than one reason.

1. The patient’s right to know their personal medical information was mentioned as one reason. As one participant described it, “*I think I have that right. It is from me; it is my body. And I know that I would rather know, otherwise it is useless to participate, I think [...]”* (Woman, 63 years old). This reason was the least frequently mentioned.
2. Another reason mentioned was that participants were simply curious. One participant indicated that he was fascinated but did not elaborate on what he found interesting or fascinating about knowing his IRR.
3. Another reason participants mentioned was that some had one or more family member(s) with dementia or who were diagnosed with AD. Since they had witnessed relatives who had AD, they knew firsthand what the disease does to a person, and this motivated them to volunteer for the study and to opt in for receiving their IRR. This reason was mentioned more frequently than the first two reasons.
4. The fourth reason was to get information so that they could Inform others about their current health situation and discuss with them how their health might change in the near future. Participants mostly referred to their partner, children, relatives, or sometimes the close environment, like their neighbors, as “others.” Participants viewed this reason for wanting to know their IRR as being an advantage. This aspect of knowing one’s IRR will be discussed more in a subsequent section.
5. Not knowing what was going on led to certain doubts, questions, and sometimes even anger, when forgetting something repeatedly. They hoped that getting their IRR would ease their mind, helping them to regain their composure and not get so visibly upset. This reason was mentioned as frequently as reasons 3 and 4 for wanting to know their IRR.
6. Five participants mentioned that they considered it a better option to receive the possibly bad “news” contained in their IRR at an early, beginning stage of a disease than to receive it at a later, advanced stage.
7. One of the most frequently mentioned reasons for participants desiring to know their IRR related to possible treatments (Fig. 1). That is, if they knew their IRR, they might be able to make better choices about treatments for the symptoms they were experiencing. Treatment was described in two ways. On the one hand, participants talked about medication that might slow down the progression of the disease and thus possibly prolong their quality of life. On the other hand, they referred to treatment as making renewed health-related lifestyle changes. For example, a participant said that he would stop his weekly habit of drinking a pint of beer, and another participant was considering taking food supplements, if this was recommended by clinicians to treat their symptoms.
8. Making informed future life decisions was the most frequently given reason for wanting to know one’s IRR. Participants referred to the opportunity of making arrangements or planning for the future, as they wanted to arrange their affairs before their memory problems became worse. Some participants clearly indicated that they had always made their own decisions, so they wanted to continue doing such, so they could avoid becoming a burden to others. The nature of this reason was diverse and could be divided into five general topical areas (Fig. 2). These will be considered in turn (a-e, below).

- Topical area (a). This area was related to making lodging accommodations. One participant mentioned how he was currently considering selling his house and moving into a smaller house or apartment.
- Topical area (b). This area was related to rearranging household tasks. Areas (a) and (b) were mentioned equally as often.
- Topical area (c). The importance of arranging for elderly residential care in advance was another topic related to making advanced arrangements. One participant explained this by describing the difficulties related to “placing” or “forcing” elderly people to go to an elderly care center against their own wishes.
- Topical area (d). Another given arrangement was to make financial decisions, such as donations to avoid inheritance taxes, or to find a person who can help with finances when the patient is no longer capable of caring for it by himself.
- Topical area (e). The most frequently mentioned topical area related to making arrangements about euthanasia. Participants said this was mostly because they wanted to relieve the burden placed on their partner and children for caring for them in a demented condition. They also did not want “*to live like a plant,”* as they often described it. While some participants had already discussed this with their partner, and sometimes with their children, some participants stated that they would discuss this only when the result was known. The participants who had already talked about this felt understood and supported by their partner and/or children. This was nuanced in two different ways:
  The first nuance related to a clarification that the request for euthanasia was not just to relieve them and their family of the consequences of AD, but also for other diseases that are legally accepted conditions according to the euthanasia law in Belgium.
  The second nuance emerged from a statement by another participant, who said that receiving a positive brain amyloid PET scan would not result in panic and the immediate consideration of euthanasia. In addition, we noticed that most of the participants were unaware of the explicit details of Belgium’s Euthanasia law and the various types of documents required for early care-planning and end-of-life decisions. This lack of information resulted in participants describing euthanasia in general terms and referring to “the document” without further clarification. Only one participant clearly described how he had all the necessary documents ready, as he had already consulted the Levens Einde Informatie Forum (LEIF).^1^ LEIF is a national forum supported by the Belgian government concerning end-of-life decisions and dignified dying. While some participants had clear ideas about which specific arrangements they would make, other participants talked in general terms about making future arrangements. When asked to elaborate, they did not know yet which arrangements they would make.

(9) Finally, the most frequently mentioned reason of our participants for desiring to know one’s IRR was to be informed about what was going on with one’s health (Fig. 1). In this vein also, participants often talked about future perspectives. If they could know what was going on, then they might know a little bit more about whether their symptoms could become worse or not.

### Perceived Advantages of Knowing one’s IRR

While we wanted to better understand why participants want to know their brain amyloid PET scan results, we also wanted to know what advantages or benefits participants perceived for IRR disclosure of brain amyloid PET imaging.

**Fig. 3:**
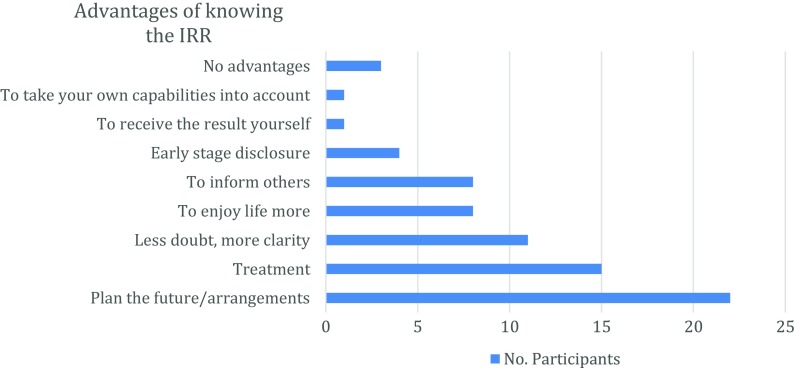
Frequencies of perceived advantages of knowing the IRR by participants

A minority of participants pointed out that there were no advantages, or at least none that they could not think of, for knowing their IRR. A participant wondered more specifically whether receiving a positive amyloid PET scan result could be perceived as advantageous. As she explained, *“[…] will there be any advantages? That’s the problem. If it is bad, is it actually, maybe disadvantageous that I know it”* (Woman, 81 years old). The majority of participants reflected on the advantages of getting their IRR. Eight advantages were mentioned, as indicated in Fig. 3. These will be considered in turn.

1. Knowing the result implies that one can take stock of one’s own capabilities and then determine what is still possible to do or not to do, according to one’s current health situation. Few participants mentioned this advantage.
2. Receiving the result yourself rather than a family member, who may or may not disclose it. One participant mentioned that if the result were disclosed to her son, for example, she was afraid that he might not give her the complete information. When asked why this would be the case, she said that her son might phrase the news in a softer way to spare her emotionally. However, she wanted to know her situation firsthand, not filtered through a third party (e.g., caregiver or family member). That is why she perceived this as an advantage, whereby the researcher-neurologist would directly and completely disclose the news to her.
3. Participants said that it would be advantageous to know the IRR at an early stage, so that they could benefit from an early intervention of treatment or imposition of health-related lifestyle changes. The difference between this advantage and the advantage of treatment (i.e., Advantage 7, below) is that participants specifically referred to the benefit of knowing this result at an early stage.
4. Compared to just giving a reason for IRR disclosure (i.e., previous section on informing others), more participants mentioned “informing others” as an advantage for knowing their IRR. While some participants focused on just informing their partner and children, other participants would also involve close friends. The benefit of informing others about their result is that they can take your new situation into account in personal interactions. One patient clearly indicated how she would tell her partner and children, but not tell anyone else. She explained, *“No, you often think that you have friends, but behind your back […]. Nowadays, people are so dastardly”* (Woman, 72 years old). By contrast, one patient expressed that she was not sure whether to inform her children, because she was afraid of hurting them. As she described it, *“Yes, I don’t even know whether or not I would tell that to my children. No, because that must be devastating if they know that their mother within 5 years... Yes, I believe I would not say it”* (Woman, 63 years old). We have to be clear that she did not refer to this aspect as an advantage or a disadvantage for knowing her IRR. This contrasting reaction does reveal how other participants can have different motivations for choosing not to inform others.
5. Some participants stated that with the knowledge of their brain amyloid status they could enjoy life more. For example, they could travel more and do the things they had wanted to do in the past but had to postpone. Although, it was not immediately clear for every participant what he would enjoy in life more. They thought this would become clear and more obvious once they know the result. One participant already started to enjoy life more since he started to experience subtle memory complaints. As he described it:

*“On the contrary, if you know something, you become more aware of this fact. Buddy, you need to enjoy life more. You know what we did? We reserved three city trips for this year. I found that; thank you, Alzheimer [laughs] […]”* (Man, 68 years old).

**Fig. 4:**
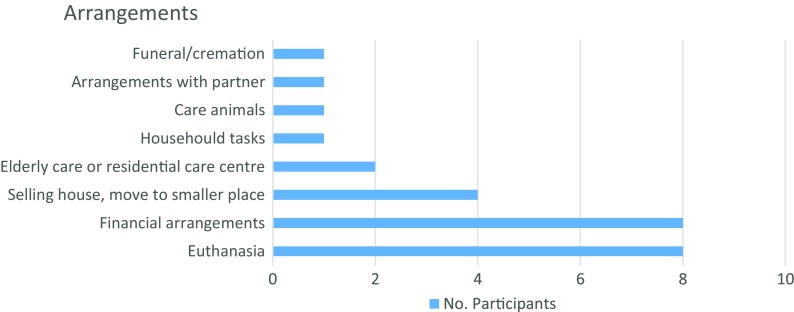
Frequencies of arrangements related to advantages mentioned by participants

(6) With new knowledge about their health, participants felt doubt was removed about what was causing their memory problems. A positive brain amyloid test result implied for some that they had achieved more clarity about their current situation:
*“[…] An advantage is, I think personally, you are with this in your mind [whether you have brain changes leading to AD]; you doubt”* (Man, 66 years old).
  Sometimes, one’s doubt is amplified when others comment on your memory problems:
*“I know that something is going on, but what? Is it Alzheimer? Is it dementia? Or is this normal with age? Yes, then they say: ‘Yes, but those people are also so old and they know more.’ But I think that it is not the same with everyone. I don’t know. It is a big question mark for me, and that’s why I want to know it”* (Woman, 68 years old).
  For some patients, no result could even lead to them losing composure, making them angry or frustrated every time they forget something.
(7) The possibility of being eligible for treatment. Patients did not only refer to treatment as a kind of medication or lifestyle changes but also to the advantageous consequences of receiving treatment, specifically follow-up medical consultations.
(8) The last advantage relates to being better prepared to plan for the future and make personal arrangements should they receive a positive result (Fig. 4).

New practical arrangements were expressed by the participants that were not mentioned earlier as reasons for wanting to know their IRR. First, arranging for one’s funeral or cremation was mentioned: *“How I want my funeral or cremation and so on. These kind of things”* (Woman, 68 years old). Another participant referred to making arrangements with their partner about creating schedules and stable places to make life easier in case her symptoms became worse. For example, she wanted to arrange for a consistent place to leave her keys and money. Another final arrangement one participant mentioned was finding appropriate care for her pets, as she is currently living alone.

One participant had a different opinion of what sort of future arrangements she should make if she knew her IRR. Although she described making arrangements for the future as being an advantage for knowing one’s results, unlike other patients, euthanasia and arranging her funeral were not included as part of her arrangements. This example shows how patients can have different ideas about what sorts of things to consider when planning for their future. For one patient, planning for one’s funeral, for example, is an absolute necessity, while for another patient, it is not so important. This sentiment is reflected in the following quote from this woman:

*“And yesterday, yes, that’s maybe, I don’t know, all the children were there, and then I told them. So, if the time comes that I have Alzheimer’s, and that they will investigate now, then I don’t want euthanasia. I have already told that to them. I want to stay in this world. I don’t know if that is an unhappy world. My mother [who had dementia] cried a lot. That won’t be so easy. But still, I don’t want euthanasia. And they can place me in the same department in that elderly care house where my mother was. Like that we talked about it. And yes, they didn’t seem surprised. But now I won’t talk about it anymore. That was that one time. But with that I know, because death and arranging your funeral, that not, that I won’t do”* (Woman, 63 years old).

In the previous section, participants talked about requesting euthanasia because they did not want to become a burden for their family or because they did not want to live in a vegetative state. While this participant indicated that having AD is most likely not an easy thing to experience, she felt that having AD does not necessarily mean that one would be living in an “unhappy world.”

### Perceived Disadvantages of Knowing one’s IRR

**Fig. 5:**
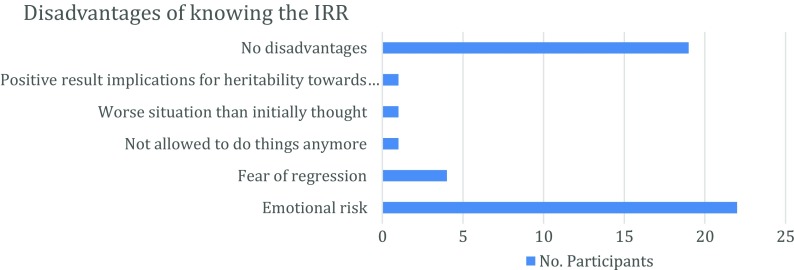
Frequencies of perceived disadvantages of knowing your IRR by participants

Next, we sought to determine whether participants perceived any disadvantages in knowing their IRR. Half of the participants spontaneously stated that there were no disadvantages related to receiving an IRR. Some indicated that disadvantages likely exist, but the advantages of knowing the results outweigh all possible disadvantages. Participants did bring up, however, five specific disadvantages associated with knowing one’s IRR (Fig. 5).

1. One disadvantage had to do with the hereditability of AD and how it would affect relatives. One participant clarified this, stating that a positive test result could be disadvantageous for her son. However, she also elaborated on this aspect, stating that it is always better to know these things in advance.
2. A second disadvantage was linked to one’s own perception of memory loss. This notion is in line with what another participant stated: Knowing the results might be disadvantageous if your results are worse than how you perceived your own subtle memory complaints to be and to how you thought your health situation was.
3. A third disadvantage mentioned was that knowing one’s results could limit one’s freedom to do certain things, like drive a car, one activity that would likely be disallowed. As one participant described it:
*“[...] Or [that] you cannot drive the car anymore, because they recently said: ‘Do you have troubles with that? Do you sometimes forget your way? Don’t you forget where you are? […] I can still do that, but I would not drive to the South of Spain. […] I would, but I don’t think my wife would like to drive with me anymore [laughs]”* (Man, 67 years old).
  Furthermore, this man indicated that if his driver’s license were taken away, this would have serious implications for his and his wife’s mobility, and this would force them to live somewhere else.
4. The fear of regression was also perceived as a disadvantage of knowing one’s IRR result. That is, the fear you get when you receive a positive test result, that your cognitive decline will become worse. Not knowing how long it will take before the progression will accelerate, leads to fear and questions for these participants.
5. A final disadvantage mentioned was the emotional risk and impact of receiving an IRR. Participants used phrases such as, “*to shed a tear, feeling worried, fear and more frustration,*” to describe the emotional impact of receiving a positive brain amyloid PET IRR. For example, one participant described it as: *“Of course it would be a shock if you heard it: You’ve got Alzheimer’s or the chance is very high that soon you will develop Alzheimer’s”* (Man, 81 years old). One participant also indicated that receiving this news may lead to internal confrontation:
*“Well, in a sense, if you don’t know what the result is, then you don’t have to make any plans. Once you know the result, then you always got the personal, because you start to think: “Well, how long, why, what can I do about this and this.” So, there is more stress and pressure than before [getting the result]”* (Man, 66 years old).
  Others indicated that it is never a pleasant thing to hear bad news, but the only way to deal with this news is to accept it and to move on with life. This last reaction indicated that many participants were aware of the possible emotional difficulties connected to knowing one’s IRR. They expressed that they do feel capable of dealing with this information (i.e., the IRR). Some participants provided more subtle answers; for example, how knowing the result can be simultaneously advantageous and disadvantageous: *“[...] The advantage is to know it, yes. If you know it, then it is a disadvantage that you have to accept it as it is, that you cannot say, yes, I don’t want to know”* (Woman, 55 years old). In addition, not knowing the news, can be emotionally difficult too, as expressed by one participant:
*“Yes, it won’t be a pleasant thing […]. If it is bad, then it is not fun. Then your world collapses; I know that too, but, does that weigh up against walking around at home and feeling bad, because you forget everything, and it is not going anymore, and you are plodding? Well, I don’t know it [...]”* (Woman, 67 years old).
  Although the news can be emotionally difficult, some participants argued that it is still better to know the result now at an early stage of the disease so one can still take actions, from starting therapy to making personal arrangements:
*“You have to, you have to be able to absorb that information of course, but if you get that now, preventive or early, you get that information next year or within 5 years, that does not matter. Sooner or later, it will come out as positive so rather sooner than later is better, because then you can still do something about it”* (Man, 63 years old).

### Participants’ Understanding of an Amyloid PET Scan Result

#### Confusion about the Meaning of the IRR: a Positive Test Result Means Good News, Right?

Throughout the interviews it was clear that the terminology researchers used to label a positive or negative brain amyloid PET scan result sometimes confused participants. A positive test result was perceived as good news by some participants. In a similar way, a negative test result was perceived by some patients as bad news. Rather than indicating the presence or absence of amyloid, some participants thought ‘positive’ or ‘negative’ referred to the emotional valence attached to the IRR. To be clear, a positive brain amyloid PET scan indicates the presence of higher levels of amyloid than age-normal, and an increased likelihood that the aMCI patient would progress to AD.

#### Limited Predictive Value

No participant spontaneously mentioned the limited predictive value of brain amyloid PET scans as a disadvantage of knowing one’s IRR. We specifically asked participants about this, how they thought about their IRR in terms of a high or low probability of developing AD. We also related this to the less-than-100% certainty of the result (i.e., risk of false positive and false negative) and the uncertainty about how the disease progression might occur.

Participants indicated that in medicine, it is not uncommon for clinicians to speak in terms of high or low chances, or use terms of percentage risk. Some participants referred to familial breast cancer for context, in which the clinician spoke of x-percentage of risk based on family history of breast cancer, x-chance of relapse, etc. In fact, participants often indicated that medicine never uses terms of absolute certainty.

Overall, participants indicated that being told they have a high chance of their current memory problems being due to early AD was already perceived as information they would like to receive, instead of knowing nothing about their current condition. They referred to possible advantages of knowing this result, such as the possibility of early intervention and disease monitoring:

*“I continue to say that the earlier something like this disease is diagnosed, even if it is not 100 percent certain, it is still better that they do everything to prevent it than to cure it afterwards”* (Man, 63 years old).

However, three subtleties were expressed in these views. (a) Some participants mentioned that if one receives the result about progressing towards AD, yet this process turns to go slower than researchers expected it would, this was perceived as a good thing. (b) A participant mentioned that there could be a disadvantage with this type of result. He described it: *“[...] The danger could possibly exist that you, of course, start with medication, while this was not necessarily required. But I don’t know if I am estimating that correctly”* (Man, 74 years old). (c) There was another participant who clearly indicated that she would like her result and her medical condition to be told as it is, without the clinician “beating around the bush.”

Other participants thought differently about the limited predictive value of this IRR. One participant indicated that it is better not to think about the limited predictive value of the result and to have a ‘wait-and-see’ attitude towards what might unfold. Another participant felt that it was better that the researchers did not speak in terms of 100% certainty, as she found this to sound very negative. She explained that if the result is a 100% certain, this takes her hope away that her situation can still turn out positively.

#### Expected Reactions after Receiving a Positive Brain Amyloid PET Scan

We asked participants how they thought they would respond if they were to receive a positive PET scan result, implying the chances were high that in a few years they might convert to AD. Different responses, depending on the coping strategy of the participants, emerged. For some participants, they stated that it was difficult to anticipate how they would respond to this news, whilst others had no difficulties and immediately indicated that they would be emotional, leading to tears, or that this news could have a negative impact on their state of mind. Some participants indicated that they would feel somehow shocked after receiving this news. As one participant described it: *“Yes, certainly a bit shocked, because you always expect that you won’t get it*” (Woman, 68 years old). As some participants presume they will receive good news, others assume, or are convinced, that they already have AD: “*Sad. Although I realize that I’m regressing, I know it”* (Woman, 81 years old). Others saw a positive PET scan result as a confirmation of their current memory problems they are experiencing.

Most participants reacted to a positive result by responding in a solution-oriented way, which was interpreted in a broad sense by them. More specifically, they imagined solutions ranging from starting therapy to making lifestyle changes to making practical arrangements.

While most participants described their own reaction to the disclosure of a positive result, some participants were more concerned about their partner’ and children’s reaction:

*“I would find that very bad for my wife, for my children, for myself also, but yes, for myself, for myself I would find that rather sad. Do you understand the difference?”* (Man, 67 years old).

Although most participants indicated that a positive brain amyloid PET scan is unpleasant news to receive, they concluded that it is better to receive this news rather than to know nothing and have lingering doubt about their own health situation. One participant indicated that it was better to receive a positive result rather than a negative result:

*“[...] then you’ve got something to plan for and you’ve got something, you know. Because it’s sometimes more worrying than if they say there is nothing there, because then, what’s my problem due too?”* (Man, 66 years old).

#### Expected Reactions after Receiving a Negative Brain Amyloid PET Scan

Patients’ reactions to a negative brain amyloid PET scan result were less diverse and can be clustered into two types of hypothetical reactions. On the one hand, some participants were happy and relieved after receiving a negative result. These participants responded either extremely enthusiastically: *“Thank you! Well, is there any champagne? [laughs][...]”* (Man, 80 years old). Whilst others responded more subtly: *“Relieved, logically […]. But still, yes, I would keep in the back of my mind that, [inarticulate] it eventually is still possible to happen in the far future. Maybe I’m already gone by then”* (Man, 73 years old).

On the other hand, many participants were left with the question: “What is causing my symptoms?” This lead to questioning the value of the result or of further investigation:

*“Yes, then I would ask myself, how it is that I’m forgetting that much [laughs]. I would ask: ‘Is everything correct?’”* (Man, 67 years old).

## Discussion

In this study, we sought to document and better understand the reasons why aMCI patients participating in a research study were either in favor of or against the disclosure of their individual brain amyloid PET scan results and what advantages/disadvantages they perceived about IRR disclosure/non-disclosure. The two most frequently mentioned reasons for choosing IRR disclosure were to better understand their own brain health status and to be able to make informed decisions about future personal arrangements they might have to make if their PET scans were consistent with the development of AD. Emotional risk was mentioned as the primary disadvantage of knowing one’s IRR.

In the field of AD research, not a lot of research on brain amyloid IRR disclosure has been done. However, a number of studies focused in particular on the desire of participants to opt for genetic AD risk assessment [16–18]. For example, a telephone survey in a community-recruited sample concluded that 79% of respondents expressed interest in predictive genetic testing for AD [18]. Another recent survey in cognitively normal older adults by Gooblar et al. indicated that 97% of participants had a strong interest in obtaining their research results, including those related to the detection of the molecular pathology of AD, such as amyloid imaging [19]. In all previously mentioned studies only a minority of participants preferred not to know their IRR. In our study, all 38 of the aMCI patients (i.e., 100% of participants) wanted to know their brain amyloid IRR. Our sample was quite diverse demographically, varying in age from 55 to 83 years (mean 71 years; 58% men), most being married with children, and most finishing secondary school or higher. An important limitation is that all 38 aMCI patients were recruited via the memory clinic and already took first initiative to receive medical advice.

Although all of our participants wanted to know their IRR, two caveats should be mentioned about these results. One participant initially did not want to know his IRR but later on changed his mind. Another participant stated that he did not want to know his IRR but said that his wife really wanted to know. In this case, we explained to him and his wife together what this would mean practically and how they might deal with the situation in which one spouse knew the results and the other did not. With additional time to reflect, this participant decided that he wanted to know the results. We carefully queried this participant’s reasoning to determine whether he was “pushed” into his decision for IRR disclosure by his wife, or whether he made the decision independently.

This high percentage of aMCI patients wanting to receive their IRR suggests that in the near future more participants might be open to participating in research if they have the possibility of receiving their IRR. However, researchers should be aware that if the release of IRR to subjects and patients were to become commonplace, it might attract people with subjective mental complaints or hypochondriacs to volunteer for research studies, which would then negatively affect not only recruiting criteria and the recruiting process but also research results. Thus, more research is needed on how participants might volunteer for a clinical trial, but opt out of receiving their IRR. Although in most studies, this group is smaller than the group of participants who do want to know, insight into the opinions and perspectives of the people who opt out of wanting to know is equally important for the IRR disclosure/non-disclosure debate.

The predictive value of brain amyloid PET scans of aMCI patients for progression to AD in the next years has certain limitations. This is an ethical issue being debated in the general literature about IRR disclosure [20–22] and is a point highlighted in our information brochure. Our hypothesis was that the limitations of the predictive value of brain amyloid PET scans would be perceived as a disadvantage by participants for wanting to know this particular type of IRR. No participants mentioned this as a disadvantage, even though they presumably read about it in the brochure. To temper the possibility that participants were providing socially desirable answers, we asked more in-depth follow-up questions about this limited predictive value. Still, it was clear that participants did not perceive this as a disadvantage. Participants with aMCI still perceived that it was better to know if they had a high or low risk of developing AD than to remain completely “in the dark” about their current health situation. In addition, our results indicate how some participants perceived this uncertainty as a positive aspect, since it leaves open the possibility for hope. Nevertheless, accurate and clear information about IRR and its associated limited predictive value remains important in order to prevent patients from misunderstanding this information.

Another hypothesis we had was that participants would mention the limited efficacy of current treatment options as a disadvantage of knowing their IRR. This hypothesis was based on published reports suggesting that available treatment options are perceived as an ethical issue [8, 20, 22]. This was not mentioned as a disadvantage by any of our participants. When asked, participants answered that they already knew before the start of the study that there is no cure available for AD, or that there is medication available but that it can only delay progression of the disease. The latter was perceived as a better option than receiving no medication at all. Some participants referred to the situation of cancer and its treatment, in which medicine can already do a lot, but is not always able to cure the disease. Most participants maintained strong hope that in the near future the situation will change for early treatment of AD, whereby researchers can rapidly make medical progress.

The concept of therapeutic misconception is important to consider when trying to understand participants’ decisions in choosing disclosure/non-disclosure of IRR. This concept, which is often mentioned by the general literature about IRR disclosure [5, 6, 23, 24], refers to the situation in which patients often fail to distinguish between clinical care and the research setting. Participants believe the researcher is a physician who has two core ethical obligations: (1) endeavor to avoid non-maleficence (no harm), and (2) work toward beneficence (doing well). “Doing well” can be understood as taking care of the health and well-being of the patient by providing necessary therapy and drug treatments [23, 25]. In this case, patients who become participants in clinical trials often expect or have high hopes that they will receive treatment. As described in our findings, participants do perceive the option for treatment as a reason to know their IRR. Thus, the therapeutic misconception also occurred in our study. Two nuances should be addressed.

First, described in the informed consent brochure as mandatory by the International Guidelines of Good Clinical Practice (ICH GCP) [26] and explained by our researchers to the study participant, was that participants would receive cholinesterase inhibitors if they had a positive amyloid PET scan. Thus, this knowledge likely would affect a participant’s decision about IRR disclosure. For a negative amyloid PET scan, this medication would not be prescribed, because it is less effective when the underlying pathology is not related to AD. Second, the concept of treatment was interpreted widely by participants and was not limited to medication. Follow-up, disease monitoring, and imposition of health-related life style changes (e.g., taking food supplements, cessation of drinking, etc.) were also thought of as treatment. This broad understanding also likely affected participants’ decisions about IRR disclosure.

The majority of our participants reflected on planning for the future as an advantage of knowing their IRR. This finding is in line with other AD studies, such as the Risk Evaluation and Education for Alzheimer’s Disease study (REVEAL) who reported on the importance of making arrangements for the future [16, 27, 28]. In our study, almost one-in-five participants also mentioned end-of-life decisions as playing a prominent role in their reasoning about IRR, more specifically the request for euthanasia, given a positive brain amyloid result. The participants who mentioned this possibility in our study might have been influenced by ongoing legal discussions in Belgium. First since 2002, euthanasia can be legally requested in Belgium under strict circumstances (Belgian Euthanasia Act, 28th of May 2002) [29]. These circumstances, imply that: (1) the patient is legally competent and conscious at the moment of making the request; (2) the request is voluntary, well-considered and repeated, and is not the result of any external pressure; (3) the patient is in a medically futile condition of constant and unbearable physical or mental suffering that cannot be alleviated, resulting from a serious and incurable disorder caused by illness or accident (section 3, §1) [29]. Second in past years, there has been a growing awareness in Belgium that healthcare institutions bear the responsibility for translating these legal regulations into optimal care for patients requesting euthanasia. For example, enhancing communication toward patients and their relatives about end-of-life decisions and early care planning [30, 31]. To properly compare the situation in Belgium with that in other countries, research is needed to evaluate whether euthanasia might be considered as a reason for wanting to know one’s IRR, and if this would lead to an increase in euthanasia requests.

aMCI participants in our study mentioned euthanasia as a practical arrangement; two ethical concerns emerged from this. First, most participants explained how they do not want to become a burden to their family members. Thus, euthanasia was perceived as a solution, whereby family members would not be burdened with a beloved one who has AD. A person’s source of “burden awareness” is an important consideration: In a euthanasia request, did the patient independently arrive at the awareness that he may become a burden, or did he become aware of it by discussions with relatives? According to the Belgian Act on Euthanasia, the request needs to be voluntary, well considered, and not the result of any external pressure [29].

Although there is a worldwide increase in the burden of caring for people with dementia, the solution for this problem should not be euthanasia but proper help throughout the care process from the perspective of both patients and caregivers. Government and healthcare regulations ought to (a) invest in damping down the perception that patients feel they are or will become a burden to their family members and in lifting the actual burden family members currently experience; and (b) maximize proper care that enhances the capabilities of patients and provide respite to ensure that the life of caregivers is not put “on hold” because they are providing care for their beloved one with dementia.

A second ethical concern that relates to aMCI participants in our study mentioning euthanasia as a practical arrangement is that participants feared living in a vegetative state, not wanting to “end up living like a plant.” This indicates how there is a negative perception or stigma about AD. Only one participant indicated that having AD does not necessarily mean that they will be living in an unhappy world.

Our findings also reveal how our participants are unclear about the legislation on end-of-life decisions in Belgium. This resulted in the use of the term euthanasia in general terms and showed that they were unaware of the different types of documents Belgian legislation provides. In Belgium, there are five different documents regarding advanced-care planning and end-of-life decisions [32]. Three of these documents are important, and our participants appear to be confused about them. The first one addresses negative advance declaration about the refusal of certain treatment options; the second addresses the stipulation that advance declaration regarding euthanasia is a document that has to be prepared in advance and has to be renewed every five years. Euthanasia will only be performed in case of irreversible coma, which excludes dementia. The third addresses an active request for euthanasia [32]. The above-mentioned criteria of the Belgian Euthanasia Act also need to be followed. It is clear that our participants confuse issues germane to these three documents. Many participants think that the advance declaration regarding euthanasia is sufficient if they receive an AD diagnosis. In reality, if a patient with an AD diagnosis wants euthanasia to be performed, he has to declare an active request for euthanasia at the earliest stage of the disease when he is still capable of expressing his wishes. This implies that the patient actively chooses to shorten his life by certain good months/years before cognitive deterioration becomes worse.

There was also confusion among some participants about the terminology used for positive and negative amyloid PET scans. Some patients understood a positive scan to be good news, a report with a favorable outcome for future brain health. Although the correct terminology was explained in the informed consent brochure and throughout the information session when the researcher explained the content of the trial face-to-face with the patient, we noticed that throughout the interviews, patients misused or misunderstood the terminology. If the interviewer noticed that a participant misunderstood the terminology, she elaborated on the point with the aim of avoiding the situation in which the interviewee and interviewer were talking about “different” results. Thus to clarify the terminology, we compared the amyloid test to an alcohol test: Having too much alcohol in the blood results in a positive alcohol test. In the same way, having too many amyloid plaques in your brain results in a positive amyloid test. Most of our participants understood this comparison. For patients to understand this type of research result, it is imperative to explain it clearly in a simple and correct way.

## Limitations

The current study aimed to gain insight into the motivations and opinions of patients with regard to the disclosure IRR of brain amyloid PET scans in a research context. The strength of this qualitative sub-study was its in-depth patient interviews, which enabled us to better understand their motivations, opinions, and experiences. Face-to-face interviews provide researchers the opportunity to go beyond numerical quantitation, which can overlook the real reasons behind a subject’s answers; interviews allow researchers to get a correct understanding of the interviewee’s opinions [33]. A limitation with all qualitative research, however, is that participants are always embedded in a certain cultural or societal setting, which can influence the results [33]. For example, the widely discussed views on euthanasia in Belgium could have affected our results. The views of participants presented here are based on a small population of aMCI patients in Belgium who were recruited via the UZ Leuven memory clinic. It is possible that somewhat different findings may emerge when investigating this topic in a different country or when recruiting a different study population. For example, subjects with minor memory complaints who have not undertaken the first steps to receive medical advice from the memory clinic or healthy adults being evaluated for preclinical AD. We do believe that the findings are important beyond the context of this research and can be of use for further clinical trials.

## Conclusion

Overall, this study showed that aMCI patients have clear motivations on why they want to know their brain amyloid IRR and what they perceive as advantages/disadvantages of knowing their IRR. The most frequently mentioned benefits were to achieve clarity regarding their deteriorating health, to start informed treatment options, and to plan for the future by making informed decisions about certain arrangements that may be required. Most of the participants mentioned the emotional risk as the main disadvantage, although most thought they would be able to cope with receiving bad news about a positive scan. The limited efficacy of current treatment options and the limited predictive value of the amyloid PET scan were not perceived as disadvantages. This latter outcome highlights that patients’ interpretation of what is written in the informed consent as possible risks and benefits can differ from what was intended by researchers.

## Appendix

**Appendix 1:** Inclusion and exclusion criteria for the BioAdaptAD study

Inclusion criteria	Exclusion criteria
Patients must meet all of the following inclusion criteria to participate in the study: • Patient is male or female and 55 – 85 years of age on the day of signing the consent form. • If female, patient lacks reproductive potential (two years post-menopausal or surgically sterile). • Patient has a subjective memory concern, as reported by subject, study partner, or clinician. • Patient has abnormal memory function documented by scoring below the criteria on the indicated cognitive exams: Education-adjusted ranges on the AVLT total learning or delayed recall percentage (≥ −1SD). • Patient has a Global Clinical Dementia Rating score of 0 or 0.5. • In the opinion of the investigator, the patient is in stable medical condition and is willing and able to perform study procedures. • Patient has at least six years of education or, in the investigator’s opinion, a work history sufficient to exclude mental retardation.	Patients will be ineligible for participation in this study if they meet any of the following exclusion criteria: - Patient has a history or current evidence of a neurological disorder that, in the opinion of the primary investigator, may contribute to the subject’s cognitive impairment. This includes but is not limited to: • Large-vessel stroke • epilepsy • Parkinson’s disease • progressive supranuclear palsy • Huntington’s disease • amyotrophic lateral sclerosis • multiple sclerosis • CNS infection • significant head trauma with loss of consciousness • normal pressure hydrocephalus - Patient has a history of large-vessel stroke or shows evidence of a large-vessel infarction or other focal lesions on a baseline brain MRI scan that might contribute to memory impairment, in the opinion of the investigator. - Patient has received an examination over the past year involving more than 10 mS ionizing irradiation. - Patient has a history within 6 months prior to screening visit or current evidence of a psychotic disorder or a major untreated depressive disorder. - Patient has a history of malignancy ≤5 years prior to signing informed consent. (Note: this does not include patients who have undergone potentially curative therapy with no evidence of recurrence for 1 year, and who are deemed as low risk for recurrence by her/his treating physician). - Patient has a history or current evidence of any potentially known clinically significant condition therapy, lab abnormality, or other circumstance that, in the opinion of the investigator, might confound the study results, or interfere with the patient’s participation over the full duration of the study, such that it is not in the best interest of the patient to participate. - Patient currently uses specific psychoactive medications (neuroleptics, chronic anxiolytics, tricyclic antidepressants, antiepileptics, anticholinergics, etc.). Stable dose trazodone, mirtazapine, or low-dose benzodiazepines prescribed for mild insomnia are allowed. - Patient currently uses anti-thrombotics, with the exception of acetyl salicylic acid (exclusionary for lumbar puncture). - Patient has a history of alcohol or substance abuse or dependence within the past 2 years (DSM IV criteria). - Patient is currently participating in or has participated in a study involving an investigational compound or neuropsychological measures within 30 days of signing informed consent. - Subject has any magnetizable metal prostheses, implants, or foreign objects that could pose a hazard during MRI scans.

## Footnotes

1 Translated as, “Before You Go.”
